# GEMINs: potential therapeutic targets for spinal muscular atrophy?

**DOI:** 10.3389/fnins.2014.00325

**Published:** 2014-10-15

**Authors:** Rebecca Borg, Ruben J. Cauchi

**Affiliations:** Department of Physiology and Biochemistry, Faculty of Medicine and Surgery, University of MaltaMsida, Malta

**Keywords:** GEMINs, motor neuron degeneration, motor neuron disease, small nuclear ribonucleoprotein assembly, SMN-GEMINs complex, spinal muscular atrophy, survival motor neuron, treatment strategies

## Abstract

The motor neuron degenerative disease spinal muscular atrophy (SMA) remains one of the most frequently inherited causes of infant mortality. Afflicted patients loose the *survival motor neuron 1* (*SMN1*) gene but retain one or more copies of *SMN2*, a homolog that is incorrectly spliced. Primary treatment strategies for SMA aim at boosting SMN protein levels, which are insufficient in patients. SMN is known to partner with a set of diverse proteins collectively known as GEMINs to form a macromolecular complex. The SMN-GEMINs complex is indispensible for chaperoning the assembly of small nuclear ribonucleoproteins (snRNPs), which are key for pre-mRNA splicing. Pharmaceutics that alleviate the neuromuscular phenotype by restoring the fundamental function of SMN without augmenting its levels are also crucial in the development of an effective treatment. Their use as an adjunct therapy is predicted to enhance benefit to patients. Inspired by the surprising discovery revealing a premier role for GEMINs in snRNP biogenesis together with *in vivo* studies documenting their requirement for the correct function of the motor system, this review speculates on whether GEMINs constitute valid targets for SMA therapeutic development.

## Introduction

Spinal muscular atrophy (SMA) is a primarily early-onset disorder characterized by spinal motor neuron loss and atrophy of the proximal limb and intercostal muscles. Considering that available therapies are, at best, palliative, SMA remains one of the most frequently (~1:6000 newborns) inherited causes of infant mortality. Afflicted patients have a homozygous loss of the *survival motor neuron 1* (*SMN1*) gene but retain one or more copies of a nearly identical homolog, *SMN2*. Owing to a key nucleotide substitution between the two genes, exon 7 of *SMN2* is often skipped, generating a truncated protein (SMNΔ7) that is unstable and rapidly degraded. SMA is thus the result of insufficient amounts of SMN protein and its levels are generally inversely correlated with the severity of the disease, hence making *SMN2* copy number the predominant modifier of the neuromuscular phenotype (reviewed in Burghes and Beattie, 2009; Monani and De Vivo, 2014). As we approach the twentieth anniversary since the discovery of the gene determining SMA (Lefebvre et al., 1995), promising therapeutic options in the pipeline, highlighted in this review, are a good reason to celebrate the two decades of relentless progress in our understanding of this devastating disorder.

Primary treatment strategies for SMA aim at boosting SMN protein levels mainly through pharmacologic agent-induced transcriptional activation or splicing pattern alteration of the *SMN2* gene, enhancement of *SMN2* exon 7 inclusion via antisense oligonucleotides (ASOs) or replacement of mutant SMN genes with functional copies by means of gene therapy vectors. Their effectiveness is highly dependent on targeting the relevant tissues at the appropriate time during disease progression, with recent studies indicating a high requirement in the motor system early during the course of the disease (Foust et al., 2010; Le et al., 2011; Lutz et al., 2011; Kariya et al., 2014). It is, however, worth pointing out that in view of mounting evidence suggesting that SMA may be a multisystem disorder (reviewed in Hamilton and Gillingwater, 2013), treatments with a global reach would probably translate into better outcomes. All in all, pharmaceutics that alleviate the SMA phenotype without augmenting SMN levels are also crucial in the development of an effective treatment, considering that these could be combined with primary options to enhance benefit to patients that fall outside the therapeutic window of opportunity. In this regard, restoring the critical function of SMN without modifying its levels is a logical avenue worth pursuing.

SMN is part of a macromolecular complex that also includes a set of diverse proteins collectively known as GEMINs. The SMN-GEMINs complex is indispensible for chaperoning the biogenesis of the small nuclear ribonucleoproteins (snRNPs), which are crucial for pre-mRNA splicing (reviewed in Cauchi, 2010; Workman et al., 2012). There is increasing evidence to suggest that deficiency of this function makes SMA a spliceopathy with consequences that are particularly detrimental to the motor unit (Zhang et al., 2008, 2013; Boulisfane et al., 2011; Lotti et al., 2012), an assumption that is still controversial (Baumer et al., 2009; Praveen et al., 2012; Garcia et al., 2013). Inspired by recent unexpected findings revealing a premier role for the GEMINs in snRNP biogenesis (Battle et al., 2006; Lau et al., 2009; Yong et al., 2010; Zhang et al., 2011; Grimm et al., 2013) along with *in vivo* studies that document a negative impact on the motor system following perturbation of key GEMIN proteins (Jablonka et al., 2002; Winkler et al., 2005; Cauchi et al., 2008; Shpargel et al., 2009; Borg and Cauchi, 2013), in this review we ask whether these predominant SMN associates constitute valid targets for SMA therapeutic development. Importantly, we explore lines of research that need to be pursued in order to validate this concept.

## Leading treatment strategies for SMA

Chief amongst the strategies directed at promoting exon 7 inclusion within *SMN2* transcripts is the use of ASOs, which are strings of modified nucleotides that bind to specific mRNA sequences. New generation ASOs are endowed with a chemistry that confers nuclease resistance and limit non-specific protein interactions. The most effectual molecules target an intron splice silencer sequence (ISS-N1) in the 5′ end of intron 7, thereby displacing factors that normally repress exon 7 inclusion (reviewed in Douglas and Wood, 2013; Sivanesan et al., 2013). Results in severe mouse models of SMA, which typically have transgenic human *SMN2* in an *Smn* knockout background, have been extraordinary. Indeed, the ASO's ability to restore full-length SMN expression normalized both neuromuscular function and lifespan (Hua et al., 2011; Porensky et al., 2012; Zhou et al., 2013). Furthermore, evidence indicates that ASO activity in peripheral tissues, in addition to the central nervous system (CNS) is necessary for robust phenotypic effects (Hua et al., 2011), though it remains somewhat contentious (Porensky et al., 2012). One drawback of ASOs is the lack of blood-brain barrier crossing, thus necessitating their delivery into the cerebrospinal fluid. In this respect, the most likely option is direct injection into the intrathecal space, which is a rather invasive method. Encouraging outcomes of a phase I and phase II clinical trial of ISIS-SMN_Rx_, an ISS-N1 targeting ASO with a phosphorothiorate backbone and 2′-O-methoxyethyl (MOE) modification developed in collaboration with academia by ISIS pharmaceuticals (Chiriboga et al., 2013; Finkel et al., 2014), have propelled this molecule to phase III of clinical development www.clinicaltrials.gov—NCT02193074.

Gene therapy has always been an attractive solution to treating genetic disorders through the restoration of the normal form of the defective gene. The use of self-complementary adeno-associated virus (scAAV) vectors to replace SMN and, thereby, rescue severe SMA mice has been a remarkable success story. This is in part due to the choice of scAAV-9 as a vector, considering its ability to both penetrate the brain and infect motor neurons in addition to other cell types. Furthermore, rapid transgene expression by virtue of its dimeric inverted-repeat genomic structure is a pivotal aspect of its potency. Independent groups reported that lifespan and motor ability were essentially rescued in severe SMA mice treated with scAAV9-SMN (reviewed in Mulcahy et al., 2014). Mirroring findings with ASOs, therapeutic benefits with regards to survival were greater when the vector was delivered intravascularly in contrast to intracerebroventricularly, hence, underscoring the global requirement of SMN for maximal recovery (Foust et al., 2010). Various challenges remain including manufacturing aspects, immune response-related issues and the need for repeated administration in view of the episomal nature of the designed vector. Nonetheless, considering the very promising results achieved in pre-clinical studies, this form of treatment has just entered the clinical circuit www.clinicaltrials.gov—NCT02122952, and results are expected to inform on the appropriate optimization.

Orally administered, blood-brain penetrating small molecule enhancers of SMN have been on the table long before the emergence of nucleic acid-based therapies. Although an overwhelming majority only induce a moderate increase in the life expectancy of severe SMA mice, a handful entered clinical development, and unsurprisingly exhibited poor efficacy in clinical trials (reviewed in Seo et al., 2013). Still in the pipeline at phase I, where safety at ascending doses is assessed, are two key compound classes. Quinazoline derivative RG3039, is a transcriptional activator of the *SMN2* gene through an unknown mechanism that involves inhibition of RNA decapping enzyme DcpS (Singh et al., 2008). In addition, proprietary molecules developed by PTC Therapeutics Inc. in partnership with Roche are able to correct alternative splicing of the *SMN2* gene (Naryshkin et al., 2012, 2014). It is noteworthy that both compound classes led to a dramatic improvement in the survival and neuromuscular function of an intermediate (Gogliotti et al., 2013) and a severe SMA mouse model (Naryshkin et al., 2012, 2014), respectively.

## Targeting SMN function rather than levels

Restoring the fundamental function of SMN without ameliorating its levels is not a novel line of therapeutic attack. In this respect, genetic and pharmacological modifiers that confer a striking phenotypic improvement that is not mediated by SMN up-regulation have been receiving more deserved attention over the years. Identified as the first fully protective modifier of SMA in humans (Oprea et al., 2008), overexpression of the F-actin bundling protein Plastin 3 was found to rescue motor unit defects in both zebrafish and mouse SMA models (Oprea et al., 2008; Ackermann et al., 2013). Recently, pharmacological inhibition of β-catenin signaling using quercetin was found to substantially improve the SMN deficiency-associated neuromuscular pathology across species, uncovering another promising pathway that can be therapeutically targeted in SMA (Wishart et al., 2014). Spurred by intriguing results in preclinical models (Imlach et al., 2012; Dimitriadi et al., 2013), the K^+^ channel modulators riluzole and 4-aminopyridine, are at phase II/III of clinical development www.clinicaltrials.gov—NCT00774423, NCT01645787. Results permitting, both can be repurposed for SMA considering that they are already FDA/EMEA-approved to treat alternative conditions. Fasudil, a RhoA/Rho kinase (ROCK) inhibitor that has been reported to promote a dramatic phenotypic improvement in an intermediate SMA mouse model (Bowerman et al., 2012), might receive a similar handling considering that it is presently on clinical trial for other disorders. Notably, the mitochondrial pore modulator olesoxime, developed by Trophos, is at a very advanced stage of clinical evolution and closer to being the first drug approved to treat SMA in view of the recently announced positive therapeutic outcomes following completion of a phase III trial (American Academy of Neurology, 2014 Annual Meeting). Commonly grouped as “neuromuscular protectants,” these examples of compounds and genetic modifiers seem to have a different mode of action since they most likely act on various downstream processes that are negatively impacted when SMN levels are insufficient.

Although no consensus has been reached, there is a sizable body of work that points to an indispensible role for SMN in spliceosomal snRNP biogenesis, specifically the assembly of a set of seven Sm proteins in the form of a ring onto small nuclear RNAs (snRNAs). Primarily, levels of SMN strongly dictate the capacity of cell extracts to produce snRNPs (Wan et al., 2005), which in turn influences SMA phenotypic severity (Gabanella et al., 2007) or phenotypic rescue in the mouse model (Workman et al., 2009). It is also noteworthy that maximal snRNP assembly activity in the spinal cord coincides with the highest exigencies of SMN during the development of the motor unit (Gabanella et al., 2005; Foust et al., 2010; Le et al., 2011; Lutz et al., 2011; Kariya et al., 2014). There is probably no other described non-canonical activity that displays a tight correlation between *in vitro* and *in vivo* characteristics including the reported involvement in axonal mRNA trafficking (reviewed in Briese et al., 2005; Fallini et al., 2012), which is often the second most-featured role for SMN. Axonal growth defects linked to disruption of this activity are typical on SMN deficiency in zebrafish (Beattie et al., 2007) and *ex vivo* mouse motor neuron cultures (Rossoll et al., 2003). For reasons as yet unknown such phenotypes have not been reported in *Drosophila* and mouse SMA models despite both displaying disrupted synaptic morphology and function (Burghes and Beattie, 2009; Grice et al., 2011, 2013; Sleigh et al., 2011).

Transcriptome abnormalities are the logical consequence of reduced or altered snRNP production, and recent *in vivo* studies have started to define the signature mRNA changes that are thought to bring about a collapse of the motor system in SMA (Imlach et al., 2012; Lotti et al., 2012; Zhang et al., 2013). Interestingly, restoration of *Stasimon*, a splicing target of SMN that is required for motor circuit function, corrects some but not all aspects of the SMA phenotype in animal models (Lotti et al., 2012). In this context, although this finding provides a link between a single splicing perturbation event and motor dysfunction, it highlights the anticipated probability that the entire SMA phenotype is the culmination of multiple instances of mRNA dysregulation (Zhang et al., 2013). Hence, would the identification of modifiers that act upstream of pre-mRNA splicing induce better disease amelioration? It is highly likely, a view supported by studies showing that restoration of normal snRNP levels through either injection of purified snRNPs or introduction of the *SMN^A111G^* allele, which is capable of snRNP assembly, corrects the disease phenotype in zebrafish and mouse SMA models (Winkler et al., 2005; Workman et al., 2009).

## GEMINs: disease-modifying candidates?

**Figure 1:**
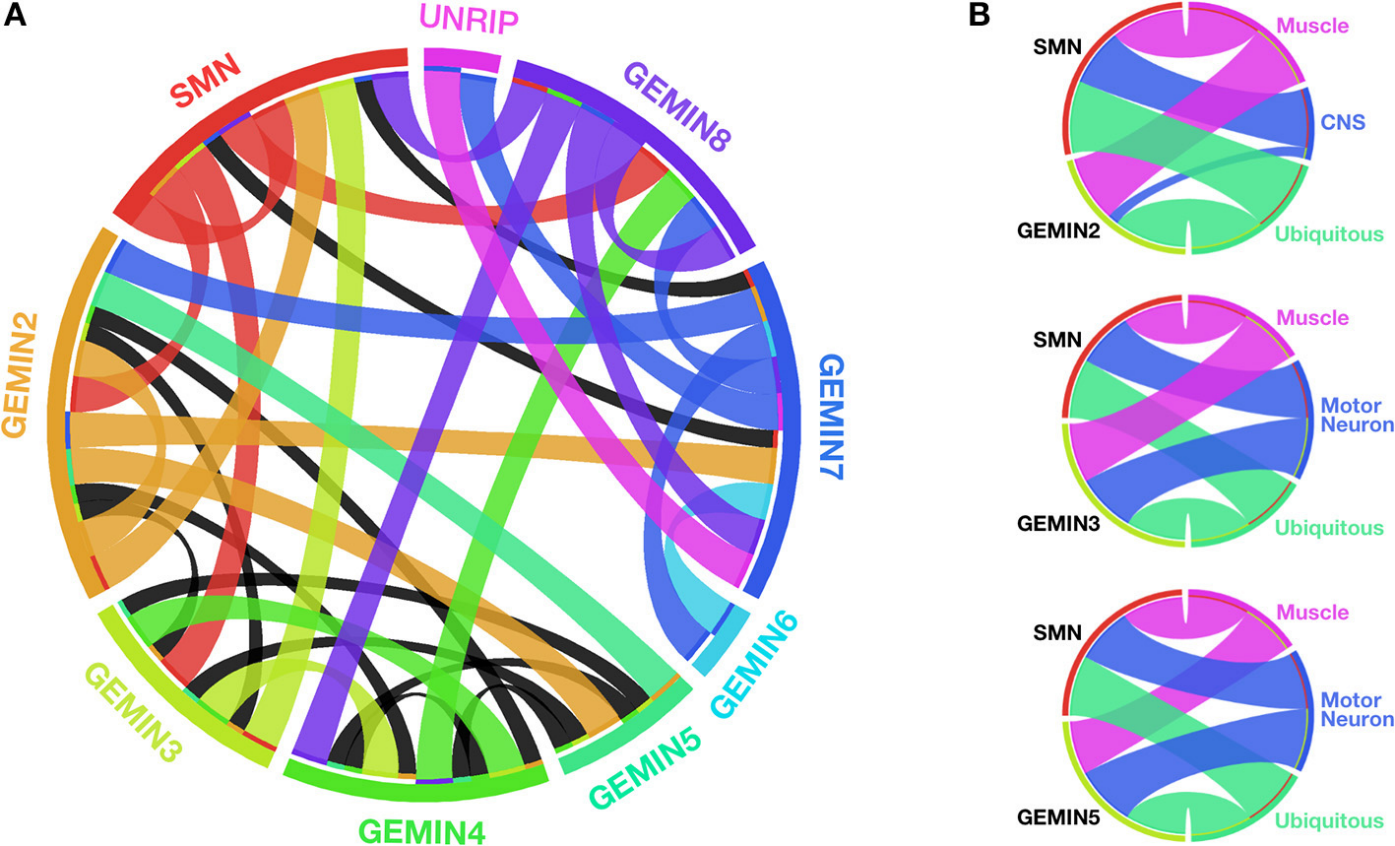
**SMN and GEMINs: protein interactions and motor system requirements**. **(A)** Circular representation of the intricate web of interactions between members of the SMN-GEMINs complex in vertebrates. Ribbons shown in colors specific to each complex member indicate interactions verified in more than one experimental system. Black ribbons specify interactions in only one experimental system (based on data reviewed in Cauchi, 2010). **(B)** Degree of overlap between specific Gemins and SMN with respect to organismal viability on enhanced RNAi-mediated knockdown starting early during development in *Drosophila* (based on data featured in Borg and Cauchi, 2013). With regards to SMN, the N4 RNAi strain developed by Chang et al. (2008) was utilized for comparison. With the exception of Gemin2 within the CNS, there is a similar requirement for both Gemins and SMN in the motor system. Width of the ribbons is inversely proportional to viability (the wider the ribbon, the lower the viability). Ribbon color corresponds to the tissue in which knockdown is restricted. Graphics in **(A,B)** were built using Circos (Krzywinski et al., 2009).

Spliceosomal snRNPs function in the nucleus, whereby in concert with numerous cofactors, they catalyze the removal of introns from pre-mRNAs, a process that is essential for the production of functional proteins. The central step in the snRNP biogenesis cycle takes place outside the nucleus, most likely to prevent partially assembled RNPs from interacting with their substrates. Key events within this cytoplasmic phase have been recently redefined to essentially shift the focus of attention from SMN to the GEMIN constituents of the SMN-GEMINs complex, which was long known to be a critical chaperone of this pathway *in vivo* (Cauchi, 2010; Li et al., 2014; Matera and Wang, 2014). Vertebrates have the most elaborate version of the SMN-GEMINs complex, formed from a web of intricate interactions between its members that include SMN, GEMINs 2–8 and UNRIP (Cauchi, 2010) (Figure 1).

Following transcription and nuclear export, the RNA arm of snRNPs is identified by free cytoplasmic GEMIN5 subunits through the stringent recognition of a code formed of sequences and structural motifs (Battle et al., 2006; Yong et al., 2010). Binding is mediated by GEMIN5's WD-repeat domain, a ubiquitous motif that gained an RNA binding label only recently (Lau et al., 2009). The majority of Sm proteins, the core protein component of snRNPs, are bound directly by GEMIN2, which wraps itself around the entirety of a crescent-shaped pentamer formed of Sm D1/D2/E/F/G. GEMIN2's reach is so remarkably extensive that it also blocks the RNA binding pocket on the Sm pentamer, hence preventing promiscuous RNA binding (Zhang et al., 2011). Interestingly, amongst the SMN-GEMINs complex members, GEMIN2 is the most phylogenetically conserved, followed by SMN, GEMIN5, and GEMIN3 in that order. Other complex components were added later during evolution, and are thus only present in Metazoans (Cauchi, 2010). These considerations underline Sm protein recognition and capture as the pivotal proofreading activity in snRNP biogenesis.

Prior to nuclear import, steps leading to ring closure through addition of the Sm B/D3 dimer, followed by the uploading of a heptameric Sm core around a conserved uridine-rich sequence of each snRNA (Sm site), are still unclear including the involvement in precise detail of other key members of the SMN-GEMINs complex. The withdrawal of SMN's participation as the primary architect of snRNP assembly raises questions about its exact role in this remarkable engineering feat. Considering that some have snRNP-independent functions (Cauchi, 2010), does SMN act as a magnet to attract the diverse members of the SMN-GEMINs complex? (Box 1) Is this property the reason why SMN has been reported to interact with a myriad of proteins (Rossoll and Bassell, 2009)? If so, what are the key chaperones that favor association with the bona fide members of the SMN-GEMINs complex, and, hence, discriminate against non-specific partners? Nonetheless, the SMN oligomers at the core of the SMN-GEMINs complex might also provide the platform on which snRNP assembly is engineered by the GEMINs.

Box 1 **Outstanding Questions**

**■ How is the SMN-GEMINs complex assembled and what are the principal chaperones involved in this process?** Although great strides were made in understanding the involvement of the SMN-GEMINs complex in snRNP assembly, little is known about how this large macromolecular machine is itself assembled. Molecular and real-time cell imaging studies will be crucial in delineating this process and identifying novel factors that provide chaperoning activities.

**■ Does up-regulation of Gemins confer a convincing strong suppression of the SMA phenotype in animal models?** Evidence favoring modulation of the disease-associated pathways through supplementation of Gemin protein levels has been sparse, and *in vivo* genetic studies are essential to address this issue conclusively.

**■ Can genetic and small-molecule therapeutic candidates that improve snRNP assembly independent of increasing SMN levels be identified?** High throughput screens are not new in the SMA field, hence technology and knowhow is already existent. The application of an SMA-associated phenotype that is directly linked to perturbation in snRNP assembly will be a fundamental issue in the design of such screens, which hold the promise of uncovering novel SMA therapeutic targets and candidates.

The highlighted molecular and structural studies provide a compelling case for the candidature of GEMINs as modifiers of SMA, presumably through suppression of defective snRNP synthesis, which is the hallmark consequence of SMN paucity. Strong support is provided by *in vivo* studies (Table 1). To this end, similar to SMN, complete loss of Gemins is unsurprisingly incompatible with life, a finding that stresses the importance of splicing to the correct functioning of an organism. Specific ablation of Gemin levels in either muscle or motor neurons results in more or less the same organismal viability profile in *Drosophila* as that observed for SMN (Chang et al., 2008; Borg and Cauchi, 2013) (Figure 1). In mice, decreased levels of *Gemin2* in an *Smn^+/−^* background induce an enhanced motor neurodegenerative phenotype that correlates with disturbed snRNP assembly (Jablonka et al., 2002). Interestingly, restricted Gemin knockdown in the motor unit was found to have a negative impact on *Drosophila* motor ability, hence resulting in phenotypes that mimic those uncovered on SMN deficiency (Chan et al., 2003; Rajendra et al., 2007; Cauchi et al., 2008; Chang et al., 2008; Grice et al., 2011, 2013; Borg and Cauchi, 2013) (Table 1). It is intriguing to note that a flurry of recent reports have uncovered disruption of Gemin protein levels and depletion of their associated nuclear bodies, known as gems, in disorders other than SMA, including amyotrophic lateral sclerosis (ALS), which is the most common adult-onset motor neuron disease (Rafalowska et al., 2014 and reviewed in Cauchi, 2014). Undoubtedly, these findings consolidate the link between GEMINs and neurodegeneration.

**Table 1:** **Phenotypes in Gemin-mutant multicellular model organisms**.

**Component**	**Organism**	**Phenotype on perturbation**	**References**
Gemin2	Worm	RNA interference-mediated knockdown results in embryonic and larval lethality	Burt et al., 2006
	Fly	Global loss of function is lethal whereas on restriction to muscle, it abrogates locomotor and flight ability	Borg and Cauchi, 2013
	Zebrafish	Antisense morpholino knockdown of Gemin2 reduces survival in embryos; conflicting reports on motor axon outgrowth phenotypes	Winkler et al., 2005; McWhorter et al., 2008
	Mouse	Homozygous knockout results in early embryonic lethality; double heterozygotes for *Smn* and *Gemin2* null alleles have enhanced motor neuron degeneration	Jablonka et al., 2002
Gemin3	Worm	Complete loss of function induces larval arrest whereas partial loss of function leads to viable organisms that have variable defects in oogenesis and progeny that is embryonic lethal	Minasaki et al., 2009
	Fly	Organisms with a global loss of function have reduced motor activity and neuromuscular junction defects prior to death at larval and/or prepupal stages; specific loss of function in the CNS or muscle confers loss of mobility and a flightless phenotype; ovarian disruption results in egg polarity defects, oocyte mislocalisation, abnormal chromosome morphology and disruption of cellular bodies	Cauchi et al., 2008; Shpargel et al., 2009; Cauchi, 2012; Borg and Cauchi, 2013
	Mouse	Homozygous loss of gene function is embryonic lethal; heterozygotes have minor defects in ovarian morphology and function	Mouillet et al., 2008
Gemin5	Fly	Organisms that are homozygous for loss of function alleles have delayed development and are larval lethal; ablation of protein levels in CNS or muscle has a negative impact on motor behavior including locomotion and flight	Gates et al., 2004; Borg and Cauchi, 2013

## Conclusions and prospects

Recent studies highlighting the prominent role of GEMINs in snRNP assembly, and the indispensability of this activity for the correct functioning of the motor system give a strong impetus for investigations that attempt at answering key open questions (Box 1). Conclusive evidence of the disease-relevance of GEMINs requires functional assessment in SMA animal models, particularly a positive impact on neuromuscular defects and life expectancy on augmentation of their function in an SMN deficient backdrop. Such proof of principle is imperative to establish whether GEMINs or any snRNP assembly enhancing candidates identified in future genetic screens constitute relevant targets for the development of therapeutic approaches that are independent of SMN supplementation. SMA-directed therapies are expected to be broad spectrum considering the emerging overlap in the pathophysiology of both SMA and ALS (reviewed in Cauchi, 2014). Although further research efforts are warranted, the possibility of reversing the course of SMA, from fatal to a treatable condition, is gathering momentum and closer than ever to becoming a reality.

### Conflict of interest statement

The authors declare that the research was conducted in the absence of any commercial or financial relationships that could be construed as a potential conflict of interest.

## Abbreviations

ASO: antisense oligonucleotide
CNS: central nervous system
ISS: intron splice silencer
scAAV: self-complementary adeno-associated virus
SMA: spinal muscular atrophy
SMN: survival motor neuron
snRNA: small nuclear ribonucleic acid
snRNP: small nuclear ribonucleoprotein.
